# Identification of a miRNA Based-Signature Associated with Acute Coronary Syndrome: Evidence from the FLORINF Study

**DOI:** 10.3390/jcm9061674

**Published:** 2020-06-01

**Authors:** Meyer Elbaz, Julien Faccini, Clémence Laperche, Elisa Grousset, Jérôme Roncalli, Jean-Bernard Ruidavets, Cécile Vindis

**Affiliations:** 1 Institute of Metabolic and Cardiovascular Diseases, INSERM UMR-1048, 31432 Toulouse, France; elbaz.m@chu-toulouse.fr (M.E.); julien.faccini@free.fr (J.F.); lacleme@wanadoo.fr (C.L.); elisa.grousset@gmail.com (E.G.); roncalli.j@chu-toulouse.fr (J.R.); 2 Toulouse Paul Sabatier University, 31432 Toulouse, France; jean-bernard.ruidavets@univ-tlse3.fr; 3 Department of Cardiology, CHU Toulouse, 31432 Toulouse, France; 4 Center for Clinical Investigation (CIC1436), CHU Toulouse, 31432 Toulouse, France; 5 Epidémiologie et Analyses en Santé publique, INSERM UMR-1027, 31000 Toulouse, France

**Keywords:** cardiovascular disease, acute coronary syndrome, biomarker, microRNA

## Abstract

Background: The discovery of novel biomarkers that improve risk prediction models of acute coronary syndrome (ACS) is needed to better identify and stratify very high-risk patients. MicroRNAs (miRNAs) are essential non-coding modulators of gene expression. Circulating miRNAs recently emerged as important regulators and fine-tuners of physiological and pathological cardiovascular processes; therefore, specific miRNAs expression profiles may represent new risk biomarkers. The aims of the present study were: (i) to assess the changes in circulating miRNAs levels associated with ACS and (ii) to evaluate the incremental value of adding circulating miRNAs to a clinical predictive risk model. Methods and Results: The study population included ACS patients (n = 99) and control subjects (n = 103) at high to very high cardiovascular risk but without known coronary event. Based on a miRNA profiling in a matched derivation case (n = −6) control (n = 6) cohort, 21 miRNAs were selected for validation. Comparing ACS cases versus controls, seven miRNAs were significantly differentially expressed. Multivariate logistic regression analyses demonstrated that among the seven miRNAs tested, five were independently associated with the occurrence of ACS. A receiver operating characteristic curve analysis revealed that the addition of miR-122 + miR-150 + miR-195 + miR-16 to the clinical model provided the best performance with an increased area under the curve (AUC) from 0.882 to 0.924 (95% CI 0.885–0.933, p = 0.003). Conclusions: Our study identified a powerful signature of circulating miRNAs providing additive value to traditional risk markers for ACS.

## 1. Introduction

Acute coronary syndrome (ACS) remains a major cause of death and disability worldwide and the number of people with cardiovascular risk factors is increasing, owing to obesity, diabetes, inactivity and ageing [1]. Currently, no high-quality soluble biomarkers are available to accurately identify subjects who are at risk of developing acute manifestations of cardiovascular diseases. Hence, there is a crucial need to discover novel biomarkers that will improve risk prediction models of ACS and help with early identification of subjects eligible for preventive treatment, as well as better stratification of high-risk patients.

MicroRNA (miRNAs) are a class of small (~22 nucleotides) non-coding RNA, which are essential post-transcriptional modulators of gene expression by binding to the 3’ untranslated region of specific target genes, thereby leading to suppression or translational repression [2]. Over the past decade, miRNAs have emerged as important regulators and fine-tuners of both physiological and pathological cellular processes, including those relevant to the cardiovascular system [3,4]. The majority of miRNAs are intracellular; however, miRNAs can also be secreted as microvesicles or exosomes and apoptotic bodies into the blood circulation. MiRNAs remain stable in the blood or serum and membrane-derived vesicles or lipoproteins can carry and transport circulating miRNAs. Indeed, miRNAs isolated from plasma are highly stable in boiling water, prolonged room temperature incubation or repeated freeze-thawing [5]. Circulating miRNAs are protected from plasma ribonucleases by their carriers, including lipid vesicles or protein conjugates, such as Argonaute 2 or other ribonucleoproteins [6].

Several miRNAs were shown to take part in the pathogenesis of cardiac injury and atherosclerosis [7] and we recently identified in stable coronary artery disease (CAD) patients a specific expression profile of circulating miRNAs as a suitable biomarker for accurate diagnosis of CAD [7,8]. In considering miRNAs are reliable biomarkers of coronary disease, the aim of the present study was to investigate the expression of plasma-circulating miRNAs between high to very high-risk asymptomatic individuals according to SCORE (Systematic Coronary Risk Evaluation) model [9] and patients who experienced an acute coronary event. We explored the association between circulating miRNAs and the risk of suffering an ACS, as well as the incremental value of a new miRNA-based signature to a clinical predictive risk model.

## 2. Experimental Section

### 2.1. Study Population

We carried out a case-control study (FLORINF, NCT02405468) recruited in the Cardiology, Arterial Hypertension and Therapeutic department of Toulouse University Hospital Center, France [10]. Cases were patients (n = 99, men and women) who experienced an acute ischemic event and were included one week after the acute ischemic phase. Demographic, clinical, and biological characteristics were recorded and a blood sample was drawn. Coronary data were available for all patients. Left ventricular ejection fraction was available at inclusion for 94 cases.

The control population (n = 103, men and women) comprised asymptomatic patients corresponding to high to very high risk primary prevention [9], including diabetes mellitus with 2 other risk factors, diabetes mellitus with chronic kidney disease or as defined by the presence of 3 of the following risk factors: treated or untreated dyslipidemia, treated or untreated hypertension, treated or untreated diabetes mellitus or current smoking. Control patients were free of known coronary disease on the basis of clinical data, echocardiogram and a systematic cardiac stress test (treadmill, echocardiography or isotopic). Exclusion criteria for both groups included the following: infectious disease within 1 week before the inclusion, immunocompromised patients, antibiotic treatment within 1 month before inclusion, chronic viral infection, chronic inflammatory intestinal bowel disease, renal failure (estimated glomerular filtration rate <50 mL/min per 1.73 m^2^) and pregnancy. Written informed consent was obtained from each patient included in the study. The study protocol conformed to the ethical guidelines of the 1975 Declaration of Helsinki and with INSERM (Institut National de la Santé et de la Recherche Médicale) and Toulouse Hospital guidelines. The study protocol was previously approved by the Institution’s ethics committee on research on humans.

### 2.2. Total RNA Isolation and Quality Control

The extraction of total RNA was performed on 200 µL of EDTA-plasma using the miRN easy serum/plasma kit (Qiagen). A synthetic *Caenorhabditis elegans* miR-39-3p (cel-miR-39-3p) miRVana miRNA Mimic (PN 4464066) was spiked into each plasma sample (5 µL aliquot of 1 nM) after the addition of QIAzol in order to monitor the efficiency of the recovery of miRNA and to normalize the expression of miRNA in the subsequent reverse-transcription quantitative polymerase chain reaction (RT-qPCR), as described previously [8]. RNA quality and quantity were measured using a NanoDrop spectrophotometer (NanoDrop Technologies, Wilmington, DE, USA).

### 2.3. MiRNA Expression Profiling

The miRNA profiles of 758 miRNAs in case (n = 6) (referred as ACS patients) and control subjects (n = 6) were investigated using the Life Technologies TaqMan Open Array Human MicroRNA panel [8,11]. Following the protocol (LTC publication PN 4470935 Rev. C) for TaqMan Open Array microRNA panel (PN 4461104), we performed on all samples a reverse transcription (RT) and preamplification steps. The cDNA was obtained with 3 µL of total RNA per sample using TaqMan MicroRNA Reverse Transcription Kit (PN 4366596) and Megaplex RT Primer Pools as we previously described [8]. Data analysis was carried out using QuantStudio 12K Flex, v1.2.2 and Expression Suite, v1.0.3; cut-off values of Ct considered as eligible were 35 and global mean normalization was used to calculate relative fold changes.

### 2.4. MiRNA Validation Phase

The expression of the selected plasma miRNAs was quantified with RT-qPCR. RT and pre-amplification were performed on all samples using custom RT primer pool and custom preamp primer pool (PN 4427975) with the protocol (LTC publication PN 4465407 Rev. B). The cDNA was prepared with 3 µL of total RNA per sample using TaqMan MicroRNA Reverse Transcription Kit and custom primer pool as described previously [8]. The normalization to cel-miR-39 was applied as described [12,13] and the values were expressed as -ΔCt. Data were analyzed on the Thermo Fisher cloud using the Applied Biosystems analysis Software v3.1.

### 2.5. Statistical Analysis

Data are presented as means and standard deviations for quantitative variables and percentages for categorical variables. The distribution of qualitative variables between cases and controls was compared using the Chi^2^ test. When basic assumptions were not satisfied, data were subjected to Fisher’s exact test. The comparison of the mean values of quantitative variables were performed by using the Student’s *t*-test. We used the Shapiro-Wilk’s and Levene’s tests to test the normality of distribution of residuals and the homogeneity of variances respectively. When basic assumptions of Student’s *t*-test were not satisfied, we performed a logarithmic transformation of the variables or a Wilcoxon-Mann-Whitney test. A forward stepwise selection was used to create a basic model. The variables—gender, current smoker, hypertension, dyslipidemia, diabetes, obesity, age and heredity—were selected a priori and stayed in the model with a retention criterion of *p* ≤ 0.10. Then, each miRNA, selected based on a case/control difference in univariate analysis, was tested in a multivariate analysis by adjusting for variables of the basic model. Logistic regression analyses were performed with polynomial models (quadratic and cubic) to examine for possible non-linear relationships between continuous variables and case-control status. Logistic regression analyses were carried out using miRNA variables categorized into tertiles. To establish the ability to differentiate cases and controls for each miRNA, the ROC curve analysis was used and the calculation of the standard error of the area under the curve (AUC) was done applying the Delong’s method. All tests were two-tailed at the level of significance of 0.05. All analyses were carried out using SAS software, version 9.4 (SAS Institute, Cary, NC, USA) and STATA statistical software, release 14.1 (Stata Corporation, College Station, TX, USA).

## 3. Results

**Figure 1:**
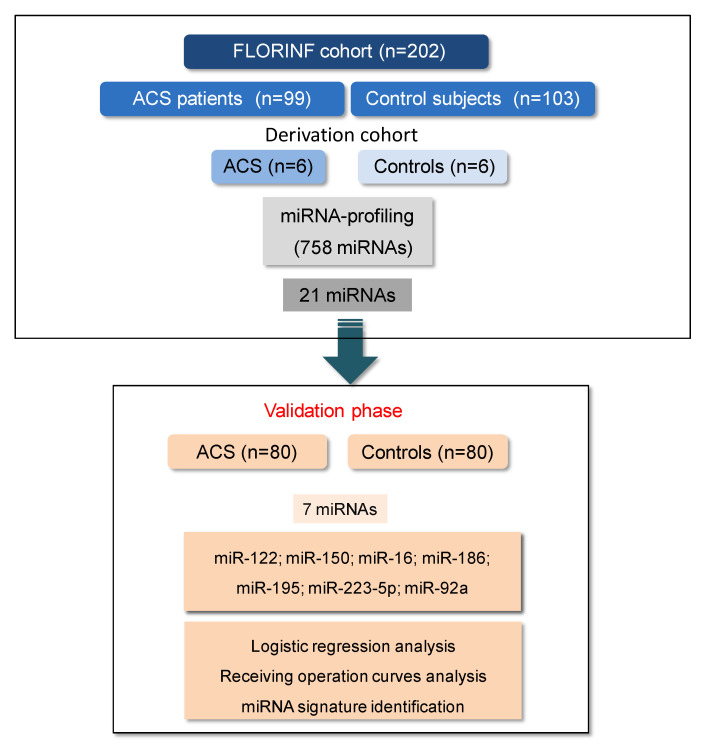
Study design and workflow. Cases (men and women) were patients who experienced an acute coronary syndrome (ACS patients) and were included one week after the acute ischemic event; controls (men and women) were asymptomatic subjects at high to very high cardiovascular risk without any coronary disease. Twenty-one miRNA candidates identified in the miRNA-profiling from the derivation cohort were analyzed in the validation phase. Nineteen samples from the 99 ACS patient cohort and 23 samples from the 103 control subject cohort were excluded because of poor RNA quality. In the validation cohort (ACS, n = 80; controls, n = 80), seven miRNAs were significantly differentially expressed in cases and controls and tested for logistic regression and ROC analyses.

The study population included ACS patients with available EDTA-plasma sample from the previously described prospective ACS-cohort (FLORINF, NCT02405468) [10]. Cases (n = 99) were patients who experienced an acute ischemic event and included one week after the acute ischemic phase (hereinafter referred to as ACS). During the same period, subjects (n = 103) at high to very high cardiovascular risk (hereinafter referred to as controls) but without any previous vascular or coronary event, particularly myocardial infarction, were recruited in the Center for the Prevention of Cardiovascular Disease in the Cardiology Department. The design of the study is described in Figure 1.

### 3.1. Screening of MiRNAs in the Derivation Cohort and Validation of Candidate miRNAs

The discovery phase of the study consisted of a miRNA microarray profile using chip-based digital PCR (758 miRNAs assessed), which was carried out on EDTA plasma RNA isolated from ACS patients (n = 6) and control subjects (n = 6) matched for age, gender, cardiovascular risk factors, and medical treatments (Table S1 in supplementary material). Data obtained from microarray analysis revealed a number of miRNAs that were differentially regulated in the plasma of ACS patients compared to control subjects. The -ΔCt values and fold changes of each detected circulating miRNA (144/758) in ACS and control groups that were up- or down-regulated are available in Table S2 in supplementary material.

Based on array results, 21 miRNA candidates with known regulatory roles in CAD were selected for prospective quantification in the validation cohort. The baseline characteristics of the 80 ACS patients and 80 control participants are summarized in Table 1.

The following 21 miRNAs were investigated in the 160 plasma samples from the validation cohort: let-7c, let-7g; miR-122-5p, miR-126, miR-133a, miR-139-5p, miR-145, miR-146a, miR-146b, miR-150, miR-155, miR-16, miR-186, miR-195, miR-21, miR-223-5p, miR-223-3p, miR-30b, miR-30c, miR-574-3p and miR-92a. Out of 21 miRNAs, 7 miRNAs were detected in all samples and differentially expressed with significance (*p* < 0.05, Table 2).

**Figure 2:**
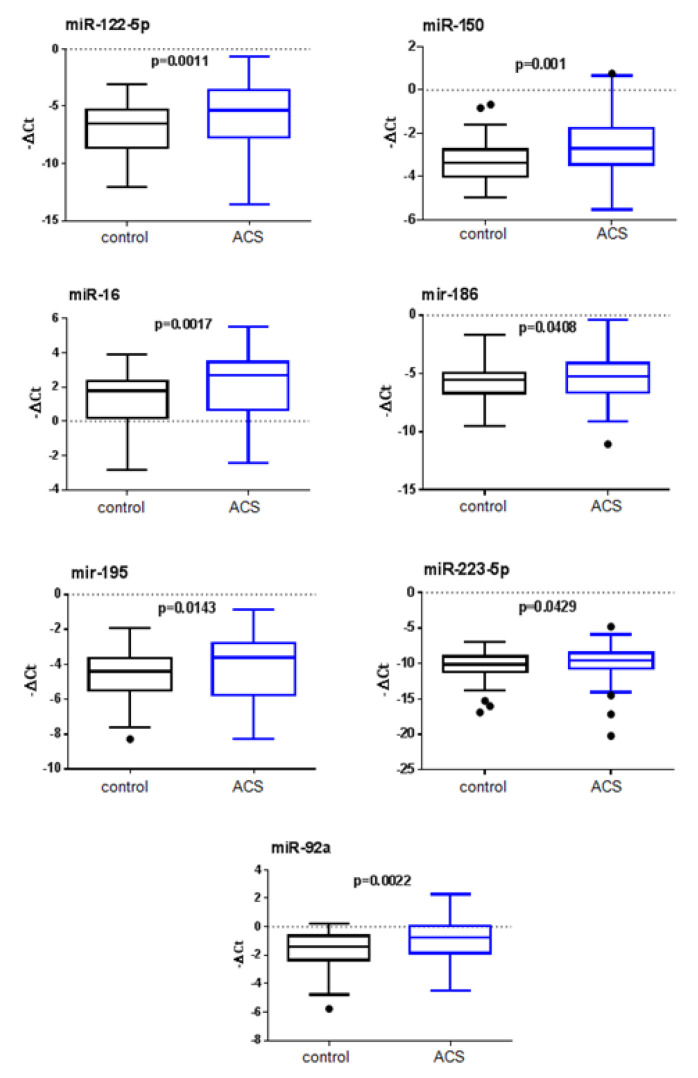
Plasma miRNA levels in the validation population. The box plots show the expression levels of miR-122-5p, miR-150, miR-16, miR-186, miR-195, miR-223-5p and miR-92a measured by RT-qPCR in ACS (n = 80) and control subjects (n = 80). The dots represent the outlier values plotted as individual points. The relative miRNA expression levels were normalized to cel-miR-39 and calculated by −ΔCt.

As shown in Figure 2, we measured significantly higher levels of miR-122-5p (referred to after as miR-122) (*p* = 0.0011), miR-150 (*p* = 0.001), miR-16 (*p* = 0.0017), miR-186 (*p* = 0.0408), miR-195 (*p* = 0.0143), miR-223-5p (*p* = 0.0429) and miR-92a (*p* = 0.0022) in ACS patients compared to control subjects.

### 3.2. Multivariate Analysis and Improved Predictive Value of the Clinical Model by Addition of the Selected miRNAs

The results from logistic regression analysis evaluating the crude estimates and adjusted associations of the seven miRNAs with ACS are presented in Table 3. For miR-122 (*p* = 0.0096), miR-150 (*p* = 0.001), miR-16 (*p* = 0.006), miR-195 (*p* = 0.0036), miR-92a (*p* = 0.0057) and miR-186 (*p* = 0.032) univariate analysis revealed that the third tertile versus the first one was significantly associated with experienced ACS, while no significance was found for miR-223-5p. After adjustment for gender, age, obesity, dyslipidemia, hypertension and smoking, the associations remained statistically significant, except for miR-186. For miR-122 (OR = 3.94), miR-150 (OR = 4.39), miR-16 (OR = 3.59), miR-195 (OR = 5.22) and miR-92a (OR = 3.31), the probability of having ACS was around between three and five times higher in the third tertile in comparison with the first one. Importantly, no significant interactions between therapeutic interventions (including statins and angiotensin II receptor inhibitors treatments that were statistically different between the two groups) and the expression level of the five miRNAs were found (Table S3 in supplementary material).

We assessed whether miR-122, miR-150, miR-195, miR-16 and miR-92a could add an incremental value to a predictive clinical model. A receiver operating characteristic (ROC) curve analysis was first performed to evaluate the diagnostic accuracy of the five selected miRNAs. As indicated in Table S4 in supplementary material, within all tested miRNA associations showing significance, the highest significant value of the area under the curve (AUC) was obtained for the combination of miR-122 + miR-150 + miR-195 + miR-16 (AUC = 0.785; 95% CI, 0.714–0.855; *p* = 0.003). However, because the miR-122 + miR-150 + miR-195 + miR-92a combination also provided a strong value (AUC = 0.778; 95% CI, 0.706–0.849; *p* = 0.002), we decided to keep miR-92a in the models.

We then tested the effects on predictive performance of adding the five selected miRNAs to the clinical model (AUC = 0.882) (Table 4). No significant *p* values were obtained when miRNAs were added individually or in pairs to the clinical model. From three up to five added miRNAs, we found that addition of, (i) miR-122 + miR-150 + miR-195; (ii) miR-122 + miR-150 + miR-195 + miR-92a; (iii) miR-122 + miR-150 + miR-195 + miR-16; or, (iv) miR-122 + miR-150 + miR-195 + miR-92a, to the clinical model resulted in statistically significant improvements (Table 4). Thereby, our data demonstrate the overall value of the selected miRNAs in the study. However, when considering AUC, 95% confidence interval and *p* value, the addition of combined miR-122 + miR-150 + miR-195 + miR-16 to the clinical model provided the best performance with an increased AUC from 0.882 to 0.924 (95% CI 0.885–0.933, *p* = 0.003). In the highest tertile for the four miRNAs, 3.8% of the control subjects and 21.3% of the ACS group were positively associated with miR-122 + miR-150 + miR-195 + miR-16. Taken together, our results highlighted a new powerful miRNA-based signature providing an incremental value to traditional risk markers for ACS.

## 4. Discussion

Circulating miRNAs are currently explored as companion biomarkers of cardiac injury, with potentially high diagnostic and prognostic powers. In the present study, we investigated the expression level of plasma-circulating miRNAs in a population of ACS patients compared to asymptomatic subjects at high to very high cardiovascular risk. We identified a new miRNA-based signature associated with the occurrence of ACS in a clinically relevant manner. The best combination of the selected miRNAs providing an additive value to the clinical model included: miR-122, miR-150, miR-195 and miR-16. This new model significantly improved the accuracy of ACS risk prediction with an increased AUC from 0.882 to 0.924, which is highly powerful when considering the clinical need of new biomarkers for identifying individuals at risk of ACS.

Based on a microarray profile of 758 miRNAs and the identification of 21 candidate miRNAs from the derivation cohort, we found significantly increased levels of 7 miRNAs in patients with ACS compared to non-ACS subjects. These data confirm numerous studies reporting deregulated levels of circulating miRNAs in patients with cardiovascular diseases [14,15,16]. Interestingly, only five miRNAs remained associated with the risk of ACS, independently of other cardiovascular risk factors. Our observations corroborate previous data revealing that differential expressions of these five miRNAs are involved in pathophysiological mechanisms related to cardiovascular outcomes. Moreover, based on ROC analysis, we retained a combination of four miRNAs, including miR-122, miR-150, miR-195 and miR-16, which provided the most powerful incremental value to the clinical model. MiR-122 is the most abundant miRNA in liver and is closely related to lipid metabolism. In human studies, miR-122 was demonstrated to be remarkably up-regulated in hyperlipidemic patients and associated with the severity of coronary heart disease [17]. MiR-122 increase was found in the cardiac tissue of patients who died from acute MI (AMI) [18] and strongly correlated with long-term morbidity and mortality in ST-segment-elevation acute myocardial infarction (STEMI) patients treated with primary angioplasty [19,20]. MiR-122 is likely involved in apoptotic and cell survival pathways since the overexpression of miR-122 in H9C2 cardiomyocytes resulted in increased caspase-3 activity and downregulation of the PTEN/PI3K/AKT pathway [21,22].

MiR-150 was associated with inflammation and was reported to be implicated in the pathogenesis of various cardiovascular diseases; however, the expression profile of circulating miR-150 in cardiac injury could vary among different studies. Indeed, Devaux et al. observed that low circulating levels of miR-150 are associated with adverse left ventricular remodeling after first STEMI [23], while circulating miR-150 was overexpressed in both STEMI and NSTEMI patients and in the early stage of AMI patients compared to control subjects [24]. Interestingly, studies indicated that miR-150 exhibited a cardio-protective role by inhibiting monocyte accumulation and cell death in a mouse model of acute myocardial infarction [25]. Indeed, the overexpression of miR-150 may directly repress the pro-apoptotic gene egr2 and p2x7r (pro-inflammatory ATP receptor) or the glucose-regulated protein-94 expression in human cardiomyocytes [26,27].

MiR-16, a member of the miR-15 family, negatively regulates cellular growth and cell cycle progression [28]. The expression of miR-16 is high in cardiomyocytes [29] and in vivo its overexpression worsened cardiac injury in a rat model of AMI [30]. On the contrary, the knockdown of miR-16 in AMI rats was cardio-protective, as evidenced by reduced cardiac apoptosis and infarct size, as well as improved cardiac function [30]. Interestingly, the β2-adrenergic receptor was identified as a target of miR-16, which may partially explain the mechanisms of miR-16’s effects on cardiac injury. Circulating miR-16 was significantly up-regulated in Takotsubo cardiomyopathic patients compared with healthy controls [31] and was positively associated with the development of left ventricular dysfunction after AMI [32].

Increased expression of miR-195, described in cardiac hypertrophy and cardiac overexpression of miR-195, resulted in heart failure in transgenic mice [33]. In addition, an inverse correlation of circulating miR-195 was observed in the presence of abdominal aortic aneurysms and increased aortic diameter, which is in line with the targets of miR-195, including collagens, proteoglycans and elastin [34]. A small simple size study of 18 patients showed that circulating miR-195 expressions (evaluated in combination with miR-30 and let-7b) were up-regulated at 8 h and 12 h after the onset of AMI symptoms and correlated with the plasma concentrations of cardiac troponin I [35]. Interestingly, in animal models of ischemia reperfusion injury [36], as well as in cardiomyocytes subjected to hypoxia-reoxygenation [37], the up-regulation of miR-195 enhanced cardiomyocyte apoptosis, suggesting that miR-195 might be a potential candidate marker and therapeutic target of AMI.

The strength and the originality of our study are based on the comparison of two well-characterized very high-risk populations that report on primary and secondary cardiovascular risk (CVR) prevention. Importantly, no interaction was found between the level of the selected miRNAs and medical treatments, thus supporting the significance of these miRNAs in ACS. Moreover, considering that miRNAs are active molecules, our study also suggests that miR-122, miR-150, miR-195 and miR-16 could represent new targets for therapeutic interventions. Additional studies are needed to determine whether differential expression of circulating miRNAs is a result of modulated intracellular production and/or different release mechanisms.

Some limitations in our study have to be taken into account. Although the sample size had enough statistical power to detect significant differences for all analyses, including a second independent cohort for validation would be useful. In addition, it would have been valuable to measure the baseline expression of the circulating miRNAs before the ACS event; however, such information required a longitudinal cohort study at high cardiovascular risk with at least 10-year follow-up.

In the perspective of our work, the monitoring of the miRNA signature both in ACS patients (secondary prevention) and in high-risk asymptomatic subjects (primary prevention) would help to determine whether the signature is a suitable companion biomarker for ACS prediction.

In conclusion, our study identified a powerful signature of circulating miRNAs providing an incremental value to traditional risk markers for ACS.

## Figures and Tables

**Table 1:** Baseline characteristics of the acute coronary syndrome (ACS) and control participants.

		ACS (n = 80)	Controls (n = 80)	*p* Value
Gender (%)	Male	82	58	0.001
	Female	18	42	
Age (years)		56.9 ± 9.4	60.1 ± 7.9	0.023
Obesity (%)		16	32	0.008
Dyslipideamia (%)		58	77	0.002
Diabetes (%)		19	24	0.86
Hypertension (%)		42	85	<0.0001
Current smoker (%)		40	15	<0.0001
Heredity (%)		29	34	0.76
Blood glucose (mmol/L)		116.8 ± 41.4	111.7 ± 58.4	0.03
Triglycerides (mg/dL)		146.1 ± 86.4	137.1 ± 70.6	0.59
Total cholesterol (mg/dL)		196.8 ± 44.5	199.2 ± 44.5	0.458
LDL-cholesterol (mg/dL) ^a^		120.1 ± 38.1	119.3 ± 40.3	0.96
HDL-cholesterol (mg/dL) ^b^		47.7 ± 11.7	52.8 ± 15.6	0.048
Medical treatment at admsission				
Beta blocker agents (%)		17	27	0.12
ACE inhibitors (%) ^c^		16	25	0.11
Antiplatelet agents (%)		17	26	0.16
Statins (%)		25	48	0.001
ARA II (%) ^d^		10	41	<0.0001
Antidiabetic treatment (%)		10	20	0.07

Data are shown as mean ± standard deviation or (%). ^a^ LDL; Low-density lipoprotein, ^b^ HDL; High-density lipoprotein; ^c^ ACE; Angiotensin-converting-enzyme, ^d^ ARA; Angiotensin II receptor.

**Table 2:** Twenty-one miRNAs selected and analyzed in the validation cohort.

	ACS (n = 80) Mean −ΔCt	SD	Controls (n = 80) Mean −ΔCt	SD	Fold Change	*p* Value
**let 7c**	−6.834	1.299	−7.06	1.247	1.270	0.2598
**let 7g**	−4.94	1.336	−4.935	1.448	0.926	0.8921
**miR-122**	−5.718	2.747	−6.9	2.261	2.258	**0.0011**
**miR-126**	−1.261	1.692	−1.521	1.401	1.010	0.395
**miR-133a**	−7.481	2.398	−7.551	2.07	1.010	0.5427
**miR-139-5p**	−6.822	1.525	−7.147	1.295	0.962	0.2484
**miR-145**	−4.728	1.552	−4.536	1.74	0.861	0.4899
**miR-146a**	−2.238	1.733	−2.523	1.637	1.057	0.3253
**miR-146b**	−4.513	1.54	−4.58	1.5	1.000	0.828
**miR-150**	−2.494	1.46	−3.311	0.847	1.575	0.001 *
**miR-155**	−7.001	1.536	−7.204	1.39	0.877	0.5137
**miR-16**	1.948	2.023	1.393	1.448	1.858	0.0017 *
**miR-186**	−5.35	1.879	−5.815	1.661	1.238	**0.0408**
**miR-195**	−4.187	1.839	−4.572	1.348	1.723	0.0143 *
**miR-21**	−3.686	1.356	−3.738	1.393	1.073	0.7035
**miR-223-5p**	−9.845	2.331	−10.34	1.864	1.510	**0.0429**
**miR-223-3p**	−0.516	2.182	−1.054	1.805	1.431	0.0825
**miR-30b**	−1.898	1.401	−1.762	1.386	0.831	0.5495
**miR-30c**	−3.239	1.626	−3.341	1.48	0.971	0.7111
**miR-574-3p**	−4.622	1.768	−5.1	1.346	1.283	0.0582 *
**miR-92a**	−1.009	1.554	−1.59	1.229	1.551	0.0022 *

The relative miRNA expression levels were normalized to cel-miR-39 and calculated by −ΔCt. SD; standard deviation. *p value* were calculated with the Wilcoxon-Mann-Whitney test; * non linear.

**Table 3:** Univariate and multivariate logistic regression analysis for the risk of ACS.

		Unadjusted			Adjusted	
	OR	95%CI	*p* Value	OR	95%CI	*p* Value
**miR-122**						
t2 vs. t1	0.68	0.31–1.50	0.34	0.79	0.26–2.37	0.67
t3 vs. t1	2.89	1.29–6.46	**0.0096**	3.94	1.28–12.1	**0.017**
Tertiles −7.0 and −5.15						
**miR-150**						
t2 vs. t1	1.23	0.56–2.70	0.61	1.17	0.38–3.64	0.79
t3 vs. t1	4.84	2.11–11.1	**0.001**	4.39	1.40–13.8	**0.011**
Tertiles −3.57 and −2.69						
**miR-16**						
t2 vs. t1	0.58	0.26–1.28	0.17	0.70	0.22–2.18	0.54
t3 vs. t1	3.48	1.53–7.91	**0.0029**	3.59	1.16–11.1	**0.027**
Tertiles 1.29 and 2.67						
**miR-195**						
t2 vs. t1	0.61	0.28–1.34	0.22	1.01	0.33–3.04	0.99
t3 vs. t1	3.39	1.49–7.72	**0.0036**	5.22	1.52–17.9	**0.009**
Tertiles −4.86 and −3.54						
**miR-92a**						
t2 vs. t1	1.15	0.53–2.52	0.73	1.72	0.53–5.61	0.37
t3 vs. t1	3.08	1.39–6.85	**0.0057**	3.31	1.07–10.2	**0.038**
Tertiles −1.73 and −0.62						
**miR-186**						
t2 vs. t1	0.64	0.30–1.41	0.27	0.61	0.20–1.86	0.39
t3 vs. t1	2.37	1.08–5.22	**0.032**	2.47	0.85–7.17	0.09
Tertiles −6.11 and −4.83						
**miR-223-5p**						
t2 vs. t1	1.08	0.50–2.32	0.85	0.94	0.31–2.84	0.92
t3 vs. t1	1.4	0.65–3.00	0.39	1.63	0.57–4.66	0.37
Tertiles −1.57 and 0						

Adjusted for gender, age, obesity, dyslipidaemia, hypertension and smoking OR, odd ratio; CI, confidence interval.

**Table 4:** Receiver operating characteristic (ROC) curve analysis comparing the predictive value of the basic clinical model with the selected circulating miRNAs added individually or in combination.

	AUC	95% CI	*p* Value
Clinical model	0.882	(0.830–0.933)	-
+ miR-122	0.903	(0.856–0.950)	0.13
+ miR-150	0.907	(0.861–0.953)	0.10
+ miR-195	0.887	(0.842–0.946)	0.30
+ miR-92a	0.900	(0.853–0.951)	0.13
+ miR-16	0.896	(0.845–0.946)	0.27
+ miR-122+miR-150	0.911	(0.866–0.956)	0.07
+ miR-122+miR-195	0.915	(0.873–0.956)	0.07
+ miR-122+miR92a	0.904	(0.857–0.951)	0.15
+ miR-122+miR16	0.909	(0.684–0.954)	0.09
+ miR-150+miR92a	0.906	(0.859–0.953)	0.11
+ miR-150+miR-195	0.909	(0.866–0.951)	0.11
+ miR-150+miR16	0.909	(0.866–0.953)	0.07
+ miR-195+miR-92a	0.906	(0.862–0.951)	0.15
+ miR-195+miR-16	0.908	(0.865–0.951)	0.10
+ miR-16+miR-92a	0.901	(0.855–0.947)	0.30
+ miR-122+miR-150+miR-92a	0.913	(0.869–0.958)	0.06
+ miR-122+miR-150+miR-195	0.925	(0.886–0.964)	0.02
+ miR-122+miR-150+miR-16	0.915	(0.871–0.958)	0.06
+ miR-150+miR-92a+miR-195	0.914	(0.872–0.956)	0.07
+ miR-150+miR-92a+miR-16	0.911	(0.867–0.955)	0.08
+ miR-16+miR-195+miR-92a	0.909	(0.865–0.952)	0.15
+ miR-122+miR-195+miR-92a+miR-16	0.919	(0.877–0.960)	0.06
+ miR-122+miR-150+miR-195+miR-92a	0.927	(0.889–0.966)	0.01
**+ miR-122+miR-150+miR-195+miR-16**	**0.924**	**(0.885–0.933)**	**0.003**
+ miR-150+miR-195+miR-92a+miR-16	0.917	(0.876–0.958)	0.07
+miR-122+miR-150+-miR-195+miR-92a+miR- 16	0.928	(0.890–0.966)	0.02

Clinical model: gender, age, obesity, dyslipidaemia, hypertension and smoking. AUC, area under the curve; CI, confidence interval.

## Supplementary Materials

Supplementary Materials can be found at https://www.mdpi.com/2077-0383/9/6/1674/s1. Table S1. Baseline characteristics of the derivation cohort, Table S2. -ΔCt values and fold changes of each detected circulating miRNA (144/758) in ACS and control groups, Table S3. Interaction of medical treatment with miRNA expression level. Table S4. Receiver operating characteristic (ROC) curve analysis comparing predictive of selected circulating miRNAs individually or in combination.

## Abbreviations

ACS	Acute coronary syndrome
AMI	Acute myocardial infarction
AUC	Area under the curve
CAD	Coronary artery disease
CVR	Cardiovascular risk
MiRNA	Micro RNA
RT-qPCR	Reverse transcription-quantitative polymerase chain reaction
ROC	Receiver operating characteristic
SCORE	Systematic Coronary Risk Evaluation
