# Supplementary material for: Identification of a miRNA Based-Signature Associated with Acute Coronary Syndrome: Evidence from the FLORINF Study
Source: J Clin Med. 2020 Jun 1;9(6):1674. doi: 10.3390/jcm9061674 (PMC7356017; doi:10.3390/jcm9061674)
Supplement: Supplementary file 1 [file jcm-09-01674-s001.pdf]

Journal of
Clinical Medicine

Supplementary Materials: Identification of a miRNA Based-Signature Associated with Acute Coronary Syndrome: Evidence from the FLORINF Study

Table S1. Baseline characteristics of the derivation cohort.

	ACS (n=6)	Controls (n=6)	*p value*
Gender (male %)	100	100	1
Age (years)	59.1 ± 5.5	59.6 ± 5.8	0.89
Diabetes (%)	0	0	1
Obesity (%)	0	16.67	1
Dyslipidaemia (%)	83.3	83.3	1
Hypertension (%)	50	83.3	0.54
Smoking (%)	16.6	16.6	1
Heredity (%)	16.6	33.3	1
Total Cholesterol (mg/mL)	192 ± 48.6	201.2 ± 26.6	0.25
LDL-cholesterol (mg/dL)	117.5 ± 47.2	122.5 ± 31.9	0.78
HDL-cholesterol (mg/dL)	47.8 ± 7.7	56.5 ± 24.4	0.96
Beta-blockers agents (%)	16.6	16.6	1
ACE inhibitors (%)	16.3	33.3	1
Antiplatelet agents(%)	16.6	16.6	1
Statins (%)	50	33.3	1
ARA II inhibitors (%)	16.6	50	0.54

Data are shown as mean ± standard deviation or n (%). LDL; Low-density lipoprotein, HDL; High-density lipoprotein;. ACE; Angiotensin-converting-enzyme, ARA; Angiotensin II receptor.

Table S2. -ΔCt values and fold changes of each detected circulating miRNA (144/758) in ACS and control groups

miRNA	ACS (n=6)		Controls (n=6)		Fold change
	Mean -ΔCt	SD	Mean -ΔCt	SD	
**000268_dme-miR-7**	-9.677	5.019	-8.305	5.092	0.386
**000377_hsa-let-7a**	-5.775	5.429	-8.303	5.966	5.768
**000379_hsa-let-7c**	-7.965	5.822	-5.625	4.685	0.197
**000390_hsa-miR-15b**	-7.377	7.006	-0.098	0.678	0.006
**000391_hsa-miR-16**	3.034	7.437	5.864	2.533	0.141
**000395_hsa-miR-19a**	-4.582	2.546	-4.371	2.816	0.864
**000396_hsa-miR-19b**	1.583	1.553	2.301	1.431	0.608
**000397_hsa-miR-21**	-7.475	5.690	-4.467	7.161	0.124
**000402_hsa-miR-24**	1.543	0.725	2.745	1.228	0.435
**000403_hsa-miR-25**	-8.146	5.152	-7.540	5.937	0.657
**000405_hsa-miR-26a**	-6.560	6.719	-0.717	2.752	0.017
**000407_hsa-miR-26b**	3.095	1.734	3.206	0.817	0.926
**000408_hsa-miR-27a**	-12.284	2.065	-9.905	4.235	0.192
**000411_hsa-miR-28**	-2.720	5.177	-1.280	1.557	0.368
**000419_hsa-miR-30c**	-13.430	1.056	-7.620	3.960	0.018
**000420_hsa-miR-30d**	-3.465	7.484	1.324	1.090	0.036
**000431_hsa-miR-92a**	1.098	2.179	2.722	2.685	0.324
**000433_hsa-miR-95**	-2.407	1.029	-6.413	5.705	16.064
**000436_hsa-miR-99b**	-4.204	6.552	0.160	0.900	0.049
**000437_hsa-miR-100**	-5.986	5.393	-7.480	6.759	2.817
**000439_hsa-miR-103**	-4.472	9.618	-2.772	8.575	0.308
**000442_hsa-miR-106b**	-5.131	5.638	-4.377	6.909	0.593
**000452_hsa-miR-127**	-8.191	5.188	-4.872	4.167	0.100
**000454_hsa-miR-130a**	-6.932	6.769	-6.778	6.208	0.899
**000456_hsa-miR-130b**	-9.656	5.145	-6.612	6.198	0.121
**000457_hsa-miR-132**	-11.388	2.503	-4.813	5.207	0.010
**000464_hsa-miR-142-3p**	-1.935	5.233	-3.890	4.890	3.875
**000468_hsa-miR-146a**	3.497	2.003	5.670	0.707	0.222
**000473_hsa-miR-150**	-2.118	5.757	0.914	7.848	0.122
**000475_hsa-miR-152**	-10.205	4.367	-6.276	5.483	0.066
**000491_hsa-miR-192**	0.693	7.135	2.677	1.266	0.253
**000494_hsa-miR-195**	-6.520	4.821	-6.953	3.389	1.350
**000497_hsa-miR-197**	-7.095	9.901	2.395	8.366	0.001
**000507_hsa-miR-203**	-9.532	6.873	-8.434	6.195	0.467
**000510_hsa-miR-206**	-11.603	4.394	-9.744	6.213	0.276

**000512_hsa-miR-210**	-2.455	8.182	-2.023	8.404	0.742
**000515_hsa-miR-212**	-7.675	6.316	-1.392	0.993	0.013
**000524_hsa-miR-221**	-2.522	4.887	-0.524	4.145	0.250
**000542_hsa-miR-326**	-12.482	2.467	-6.501	1.532	0.016
**000543_hsa-miR-328**	-4.082	9.676	5.216	1.152	0.002
**000545_hsa-miR-331**	-5.339	5.937	-3.366	4.848	0.255
**000554_hsa-miR-361**	-4.434	7.050	-2.916	5.810	0.349
**000563_hsa-miR-374**	-5.392	3.625	-7.698	4.460	4.948
**000564_hsa-miR-375**	-11.667	4.568	-6.347	5.773	0.025
**000565_hsa-miR-376a**	-11.774	3.277	-6.744	5.395	0.031
**000572_hsa-miR-382**	-9.879	4.844	-9.410	4.836	0.723
**000580_hsa-miR-20a**	-1.819	5.784	0.613	0.765	0.185
**000587_hsa-miR-29c**	-9.105	4.400	-6.259	5.901	0.139
**000602_hsa-miR-30b**	-9.190	5.878	-1.553	6.570	0.005
**001006_RNU48**	1.858	3.554	1.166	1.209	1.615
**001014_hsa-miR-20b**	-2.248	7.901	1.937	1.112	0.055
**001027_hsa-miR-432#**	-5.763	7.700	-4.864	6.662	0.536
**001028_hsa-miR-433**	-13.430	1.056	-10.230	3.698	0.109
**001090_mmu-miR-93**	2.536	7.238	3.801	0.946	0.416
**001094_RNU44**	-2.281	5.333	-1.519	0.967	0.590
**001097_hsa-miR-146b**	0.344	6.130	0.829	0.941	0.714
**001141_mmu-miR-451**	-3.173	7.214	-2.229	5.274	0.520
**001159_hsa-miR-518d**	-5.593	8.049	3.079	1.023	0.002
**001167_hsa-miR-520a**	-3.721	10.572	0.328	6.519	0.060
**001187_mmu-miR-140**	0.815	1.691	-0.137	1.361	1.935
**001278_hsa-miR-486**	-5.729	7.901	-0.209	6.862	0.022
**001285_hsa-miR-487b**	-2.564	5.296	-3.713	0.942	2.218
**001319_mmu-miR-374-5p**	-1.422	5.346	0.981	1.073	0.189
**001515_hsa-miR-660**	-9.781	4.860	-6.220	6.401	0.085
**001516_hsa-miR-425-5p**	-6.625	6.978	-5.596	6.620	0.490
**001518_hsa-miR-532**	-10.274	4.219	-5.746	3.662	0.043
**001531_hsa-miR-564**	0.244	7.119	1.414	1.449	0.444
**001541_hsa-miR-548b**	1.937	7.875	2.799	1.181	0.550
**001562_hsa-miR-629**	-5.748	5.216	-6.506	4.969	1.691
**001592_hsa-miR-642**	-5.260	8.932	0.257	1.427	0.022
**001614_hsa-miR-572**	-11.230	5.287	-4.445	7.939	0.009
**001630_mmu-miR-491**	-0.800	1.749	-1.595	1.169	1.735
**001821_hsa-miR-484**	-12.197	2.270	-5.110	7.094	0.007
**001973_U6 rRNA**	4.773	2.910	3.023	1.040	3.364
**001986_hsa-miR-766**	3.354	1.360	2.480	1.178	1.833

**001988_hsa-miR-598**	-7.421	4.466	-9.553	3.960	4.384
**001992_hsa-miR-668**	-7.487	6.318	-5.459	6.386	0.245
**001998_hsa-miR-769-5p**	6.061	2.771	5.933	1.460	1.093
**002087_hsa-miR-505#**	-3.331	1.913	-4.865	2.334	2.896
**002088_hsa-miR-636**	-6.646	4.484	-1.908	1.170	0.037
**002098_hsa-miR-223#**	-1.419	5.336	-0.177	1.177	0.423
**002099_hsa-miR-224**	-7.537	6.119	-7.824	6.818	1.220
**002122_hsa-miR-376c**	-9.986	4.635	-2.903	2.476	0.007
**002161_hsa-miR-324-3p**	1.150	1.201	0.468	1.082	1.604
**002169_hsa-miR-106a**	1.131	1.097	1.619	1.533	0.713
**002186_hsa-miR-345**	-1.266	5.878	0.101	1.310	0.388
**002187_hsa-miR-942**	0.119	6.107	-0.325	6.212	1.360
**002193_hsa-miR-886-5p**	-8.841	6.575	-4.164	4.322	0.039
**002196_hsa-miR-99b#**	-2.337	5.198	-0.128	1.582	0.216
**002215_hsa-miR-196b**	-5.771	5.760	-7.221	4.554	2.731
**002216_hsa-miR-128a**	-7.821	5.576	-10.031	5.137	4.629
**002227_hsa-miR-323-3p**	-10.066	4.557	-12.158	3.794	4.263
**002228_hsa-miR-126**	1.424	6.706	4.976	1.944	0.085
**002234_hsa-miR-140-3p**	-9.762	5.168	-1.845	2.913	0.004
**002245_hsa-miR-122**	-7.704	5.741	-7.380	7.690	0.799
**002246_hsa-miR-133a**	-9.599	5.692	-4.810	6.544	0.036
**002248_hsa-miR-142-5p**	-8.191	5.581	-6.838	5.657	0.392
**002249_hsa-miR-143**	-9.913	5.191	-8.637	5.189	0.413
**002258_hsa-miR-340**	-4.643	4.890	-4.485	1.782	0.896
**002259_hsa-miR-340#**	-0.217	1.668	-3.708	5.356	11.246
**002260_hsa-miR-342-3p**	-2.855	11.252	2.193	1.310	0.030
**002271_hsa-miR-185**	-5.458	5.597	-3.574	4.913	0.271
**002275_hsa-miR-370**	-4.109	6.968	-4.591	7.225	1.396
**002276_hsa-miR-222**	-11.360	5.303	-6.117	6.453	0.026
**002277_hsa-miR-320**	5.054	1.525	2.556	8.542	5.651
**002278_hsa-miR-145**	-2.644	4.981	-0.100	1.040	0.172
**002282_hsa-let-7g**	-0.431	5.768	0.811	0.997	0.423
**002283_hsa-let-7d**	2.279	1.454	-1.555	5.790	14.256
**002285_hsa-miR-186**	3.624	1.490	2.871	0.912	1.686
**002289_hsa-miR-139-5p**	-2.206	1.332	0.931	0.813	0.114
**002295_hsa-miR-223**	5.308	0.995	5.822	2.561	0.700
**002296_hsa-miR-885-5p**	-0.967	5.755	1.261	1.911	0.214
**002299_hsa-miR-191**	2.463	7.175	5.270	2.450	0.143
**002302_hsa-miR-425#**	0.938	4.348	-1.153	1.524	4.261

**002304_hsa-miR-199a-3p**	-10.337	4.095	-8.549	3.751	0.290
**002308_hsa-miR-17**	2.508	7.300	6.148	0.624	0.080
**002313_hsa-miR-139-3p**	-8.601	6.768	0.327	1.109	0.002
**002323_hsa-miR-454**	-6.891	6.708	-6.357	7.307	0.691
**002324_hsa-miR-744**	-1.803	5.088	-0.710	1.545	0.469
**002338_hsa-miR-483-5p**	-6.233	7.314	1.906	1.120	0.004
**002340_hsa-miR-423-5p**	-5.053	6.198	-0.363	1.333	0.039
**002349_hsa-miR-574-3p**	-5.431	5.591	-5.253	3.904	0.884
**002365_hsa-miR-494**	-6.174	6.314	-9.009	5.145	7.131
**002367_hsa-miR-193b**	-1.638	8.715	3.191	2.057	0.035
**002376_hsa-miR-543**	-3.137	5.034	-7.387	6.591	19.025
**002392_hsa-miR-301b**	-9.955	5.060	-8.940	5.542	0.495
**002400_hsa-miR-520c-3p**	3.064	9.505	9.594	3.205	0.011
**002406_hsa-let-7e**	-1.778	8.460	3.032	1.014	0.036
**002422_hsa-miR-18a**	-5.861	5.266	-4.359	4.222	0.353
**002432_hsa-miR-625#**	-2.967	10.605	-6.931	1.163	15.597
**002434_hsa-miR-628-3p**	-3.662	4.426	-5.396	3.753	3.326
**002436_hsa-miR-629**	-8.731	4.965	-9.574	3.817	1.795
**002439_hsa-miR-23a#**	4.514	2.853	3.244	1.430	2.411
**002442_hsa-miR-25#**	-9.447	5.642	-10.258	4.912	1.754
**002443_hsa-miR-26a-1#**	-9.480	4.338	-10.183	3.549	1.627
**002658_hsa-miR-338-5P**	-9.581	3.655	-10.592	3.160	2.015
**002768_hsa-miR-1233**	-9.655	5.724	-1.145	1.197	0.003
**002779_hsa-miR-1271**	-10.040	3.318	-11.760	3.384	3.293
**002838_hsa-miR-1291**	-3.668	4.956	-7.870	6.956	18.405
**002844_hsa-miR-320b**	-5.907	5.544	-5.822	3.688	0.943
**002847_hsa-miR-1180**	-8.235	5.344	-9.625	4.245	2.622
**002883_hsa-miR-1274a**	-0.787	6.233	-2.823	8.597	4.103
**002884_hsa-miR-1274b**	9.493	1.203	9.816	1.328	0.799
**002895_hsa-miR-720**	5.359	1.094	4.061	1.436	2.459

Circulating miRNAs were detected by using the TaqMan Open Array technology in plasma of ACS patients (n=6) and controls (n=6). Data are shown as relative miRNA expression levels and normalized using the global mean n ormalization method and expressed as -ΔCt. The expression fold change are expressed as 2^(ΔΔCt) in which values represent the fold change miRNA expression of ACS patients relative to miRNA expression of control subjects.

Table S3. Interaction of medical treatment with miRNA expression level.

	Statins	ARA II inhibitors
mir-122	0.35	0.96
mir-150	0.95	0.44
mir-16	0.48	0.63
mir-195	0.67	0.41
mir-92a	0.24	0.56

ARA II ; Angiotensin II receptor. The logistic models were adjusted on statins or ARA II inhibitors and a statistical interaction was sought for each miRNA analyzed.

Table S4. Receiver operating characteristic (ROC) curve analysis comparing predictive of selected circulating miRNAs individually or in combination.

	**AUC**	**95 % CI**	***p value***
MiRNAs			
miR-122	0.655	(0.574-0.736)	-
miR-150	0.665	(0.585-0.745)	-
miR-195	0.680	(0.601-0.759)	-
miR-92a	0.623	(0.541-0.706)	-
miR-16	0.688	(0.610-0.767)	-
miR-122+miR-150	0.722	(0.644-0.799)	0.08
miR-122+miR-195	0.737	(0.661-0.813)	**0.02**
miR-122+miR-92a	0.690	(0.610-0.771)	0.23
miR-122+miR-16	0.732	(0.655-0.809)	**0.03**
miR-150+miR-92a	0.678	(0.595-0.760)	0.35
miR-150+miR-195	0.743	(0.667-0.818)	**0.01**
miR-150+miR-16	0.733	(0.656-0.809)	**0.03**
miR-195+miR-92a	0.701	(0.621-0.780)	0.47
miR-195+miR-16	0.707	(0.629-0.785)	0.24
miR-16+miR-92a	0.706	(0.627-0.785)	0.11
miR-122+miR-150+miR-92a	0.726	(0.648-0.804)	**0.04**
miR-122+miR-150+miR-195	0.776	(0.705-0.847)	**0.003**
miR-122+miR-150+miR-16	0.760	(0.687-0.834)	**0.005**
miR-150+miR-92a+miR-195	0.754	(0.679-0.829)	**0.009**
miR-150+miR-92a+miR-16	0.738	(0.661-0.814)	**0.04**
miR-16+miR-195+miR-92a	0.721	0.643-0.798)	0.15
miR-122+miR-195+miR-92a+miR-16	0.762	(0.689-0.835)	**0.005**
miR-122+miR-150+miR-195+miR-92a	0.778	(0.706-0.849)	**0.002**
**miR-122+miR-150+miR-195+miR-16**	**0.785**	**(0.714-0.855)**	**0.003**
miR-150+miR-195+miR-92a+miR-16	0.757	(0.683-0.831)	**0.007**
miR-122+miR-150+-miR-195+miR-92a+miR- 16	0.774	0.702-0.845)	**0.003**

© 2020 by the authors. Submitted for possible open access publication under the terms and conditions of the Creative Commons Attribution (CC BY) license (http://creativecommons.org/licenses/by/4.0/).
